# Beyond gentrification: urban rent and foundational liveability in Southern European intermediate cities

**DOI:** 10.3389/fsoc.2026.1923781

**Published:** 2026-09-18

**Authors:** Angelo Salento, Vanessa Corrado, Carlotta Ebbreo, Vincenzo Latronico, Antonello Podda, Valeria Saiu

**Affiliations:** 1 Department of Human and Social Sciences, University of Salento, Lecce, Italy; 2 Department of Political and Social Sciences, University of Catania, Catania, Italy; 3 Department of Political and Social Sciences, University of Cagliari, Cagliari, Italy

**Keywords:** foundational liveability, intermediate cities, ordinary cities, rent gap, touristification, urban rent

## Abstract

Contemporary urban scholarship frequently interprets transformations of urban space through the category of gentrification. This article argues that, in Southern European non-competitive intermediate cities, urban rent – understood here as encompassing both land and property rent – provides a more effective interpretive lens. Through a comparative analysis of Cagliari, Catania, and Lecce, we show that intense processes of property valorization can unfold even in the absence of significant displacement and social replacement. Drawing on the tradition of Italian critical planning and sociology, we distinguish between marginal land rent – the rent generated by urban expansion through the conversion of agricultural or peri-urban land into developable urban land – and differential rent, associated with the valorization of the existing built environment. The analysis highlights the continuity between these two cycles: peripheral expansion contributed to the depopulation and devaluation of historic centres, creating the conditions for subsequent processes of selective upgrading, functional conversion, and touristification. The findings show that the rent gap tends to close primarily through the replacement of urban uses rather than the replacement of residents. The contemporary valorization of historic centres is in fact based on the progressive erosion of residential functions and their reorientation towards tourist, recreational, and commercial uses. Although these processes do not necessarily take the form of classical gentrification, they have significant effects on the foundational liveability of intermediate cities. The article therefore proposes urban rent, rather than gentrification, as the primary category for interpreting transformations in Euro-Mediterranean intermediate cities. It also shows how these dynamics are supported by a broad social coalition invested in property valorization, corresponding to the real-estate bloc (*blocco edilizio*) described by the Italian critical tradition.

## 1 Introduction

This article proceeds from three premises. The first is a claim for the analytical dignity of non-competitive intermediate cities: they are not minor objects of study or peripheral cases, but contexts with trajectories of their own. The second is that studying them requires categories different from those developed from metropolitan contexts – not scaled-down adaptations, but analytical tools constructed from the specificity of these places. The third is that such categories illuminate structural features of urban transformation that remain obscured in large urban centres by the sheer intensity of the processes unfolding there.

Contemporary urban theory tends to interpret processes of property valorization and residential transformation through the category of gentrification. From its original formulation (Glass, 1964; Smith, 1979, 1996) onwards, the concept has become one of the principal interpretive tools for urban change, often used as an umbrella category covering urban upgrading, property reinvestment and the reconfiguration of urban uses. Over the past two decades a growing body of scholarship has documented its spread along the urban hierarchy, showing that phenomena once associated almost exclusively with the large metropolises of the Global North can now also be observed in intermediate cities.

The systematic review by Kubeš and Kovács (2025), which analyses 127 publications on so-called provincial gentrification, offers a representative synthesis of this strand of research. Yet it reveals a recurrent limitation: intermediate cities are systematically described as contexts in which gentrification occurs in an attenuated, delayed or incomplete form compared with the metropolitan model – ‘somewhat delayed, less dynamic and often incomplete’ (Kubeš and Kovács, 2025, p. 1), with less affluent gentrifiers, less pronounced displacement and less extensive physical transformation. Every dimension of the phenomenon is measured as a deficit against a benchmark, the metropolis, that continues implicitly to operate as the full and normative case. Intermediate cities are thus understood primarily in terms of what they are not: not large enough, not dynamic enough, not sufficiently gentrified.

This limitation points to a broader tension that has marked the literature on urban transformation since its inception. On one side are studies that emphasize the structural mechanisms of urban valorization – rent, value differentials, and the conditions under which reinvestment becomes profitable; on the other, analyses focused on its observable outcomes – social replacement, changes in neighbourhood composition, and transformations of the urban landscape. The centre of gravity has gradually shifted towards the second pole, for reasons that are epistemic before they are empirical: the outcomes of gentrification are more accessible to the tools of culturally oriented social science than the underlying mechanisms, which require a theory of value and a sociology of rent. The descriptive level has consequently come to overshadow the explanatory one, often to the point of being mistaken for it.

In recent years, some authors have sought to overcome these limitations by redirecting attention to the institutional and political conditions that make urban valorization possible. Bernt’s (2022) concept of the gentrification gap, for example, emphasizes the importance of the institutional configurations that enable the rent gap to close and cautions against reducing gentrification to the behaviour of gentrifiers alone. This shift represents an important advance over more culturalist interpretations. Nevertheless, it continues to treat gentrification as the principal lens through which urban transformations are understood. Our proposal is different: in non-competitive intermediate cities, urban rent is not simply the background to gentrification, but the phenomenon to be studied. Gentrification is only one possible outcome of valorization and, as in the case of intermediate cities, often not the most important one.

Smith (1979) had already identified this asymmetry in the paper that introduced the concept of the rent gap: demand for gentrified housing and consumer preferences, he argued, are secondary; what drives the process is the pursuit of the difference between currently capitalized land rent and the potential rent that can be extracted through a different use of the land. Gentrification is a possible outcome, not the process itself. Subsequent scholarship has largely inverted this hierarchy – an inversion that becomes acutely problematic wherever the expected outcome does not occur, as is the case in many intermediate cities.

This article therefore proposes bringing the category of urban rent back to the centre of analysis. Our argument is that, in Southern European non-competitive intermediate cities, rent is a more powerful interpretive tool than gentrification. Whereas the latter describes a particular outcome of urban valorization, rent makes it possible to identify the mechanism that transforms space even in the absence of displacement. It operates when already depopulated areas are repurposed; when the building stock is converted to tourist uses; and when the economic and social functions of neighbourhoods change without any clear replacement of residents. In these contexts, touristification, functional conversion, and the reorganization of urban uses are fully operative forms of rent extraction, even though they do not assume the classical form of gentrification.

The analysis of intermediate cities also brings a second dimension into view. Urban rent unfolds over the long term as a cumulative process in which each cycle of valorization creates the conditions for the next: in the cities examined here, the peripheral expansion of the second half of the twentieth century contributed to emptying historic centres of residents, and it is on that devaluation that the differential rent now being extracted depends. The rent gap does not predate this history; it is its product.

The argument developed in this article forms part of a broader critique of the metrocentrism that has long characterized urban theory. Beginning with the ordinary cities approach (Robinson, 2006), numerous studies have challenged the idea that large metropolises constitute the privileged laboratory of urban theory and that other cities should be understood as incomplete or peripheral versions of them. From this perspective, urban comparison is not a means of measuring distance from an ideal model, but of recognizing the plurality of trajectories through which capitalism produces and organizes urban space (Robinson, 2011, 2016; Roy, 2009; Peck, 2015) – a critique that runs parallel to the challenge mounted by the grounded city approach against agglomeration- and competitiveness-based accounts of urban success (Engelen et al., 2017). Intermediate cities are not minor cases of urban modernity, but places where mechanisms that elsewhere tend to be concealed by the scale and complexity of metropolitan processes become visible.

The foundational economy approach (Engelen et al., 2017; Calafati et al., 2023) is aligned with this perspective, inviting us to assess cities not in terms of competitiveness but in relation to their capacity to sustain residents’ everyday lives. From this standpoint, property and territorial transformations matter not only as economic phenomena, but also because of their effects on the availability of affordable housing, households’ residual income, access to services, and the quality of social infrastructure. In other words, they directly affect cities’ foundational liveability.

It is from this perspective that the article presents a comparative study of Cagliari, Catania and Lecce. As the analysis will show, urban rent provides a more productive analytical category than gentrification for interpreting contemporary transformations in cities of this kind, and these transformations are better understood by recovering a tradition that has remained marginal in international debate: Italian critical planning and sociology, which since the 1960s has developed a theory of urban rent suited to Euro-Mediterranean regimes of valorization. That tradition allows us to trace long cycles of rent accumulation and to identify the role of an interclass socio-political formation – the real-estate bloc described by Parlato (1970) – in reproducing urban regimes hostile to the public capture of rent.

The remainder of the article is organized as follows. Section 2 develops the theoretical framework of urban rent and its regimes; Section 3 presents the cases and methodology; Section 4 discusses the findings of the comparative analysis; Section 5 examines the effects on foundational liveability; Section 6 develops the theoretical discussion; Section 7 considers the transferability of the framework beyond the Italian case; and Section 8 presents the conclusions.

## 2 Urban rent, regimes of valorization, and spatial transformation in ordinary cities

As argued in the introduction, the principal limitation of the category of gentrification is that it describes a possible outcome of urban valorization more than the mechanism that produces it. When that outcome does not occur, or occurs only in partial forms, the concept tends to lose explanatory power. The category of urban rent, by contrast, makes it possible to analyse the generative process of valorization regardless of the concrete forms it assumes (Ricardo, 1817; Marx, 1894/1981; Harvey, 1973, 1978, 1982; Lefebvre, 1974).

Treating rent as an analytical category means shifting attention from observable effects to the conditions that make it possible to extract value from urban space. Gentrification is then only one of the ways in which rent is realized. Touristification, the functional conversion of the building stock and the commercial specialization of historic centres can generate equally significant effects without intense social replacement; in non-metropolitan contexts in particular, the rent gap may be actively produced by urban governance and operate in the absence of fully developed gentrification (Slater, 2017; Holmes, 2025). The distinction matters especially in Southern European intermediate cities, characterized by relatively shallow property markets, limited financialization and a long history of demographic decline in their historic centres.

This is the point at which our proposal departs from recent attempts to reground the analysis of gentrification in its institutional conditions, discussed in Section 1: what those attempts treat as the setting in which gentrification becomes possible, we treat as the phenomenon to be explained.

Adopting rent as a category offers a second advantage. Unlike gentrification, which tends to focus on specific moments of urban reinvestment, it enables us to reconstruct the historical continuity between different phases of urban development. Contemporary opportunities for valorization do not arise in a vacuum; they are the result of previous transformations that altered the distribution of values and uses across urban space. From this perspective, rent allows urban change to be read as a cumulative, long-term process.

A particularly useful key to interpreting this continuity is provided by the Italian critical planning tradition, and specifically by the distinction between marginal land rent and differential rent (Tocci, 2009). Marginal land rent is generated by urban expansion through the incorporation of new land into the urban circuit via urbanization, infrastructure provision, and changes in land use; differential rent, by contrast, derives from the transformation, upgrading, or functional conversion of spaces that are already urbanized. Although the two forms may coexist within the same urban context, they tend to characterize different phases of city development and to operate through distinct mechanisms.

Marginal rent was the dominant form during the expansionary cycle of the second half of the twentieth century. Population growth, the outward extension of cities, and the spread of car ownership made the conversion of agricultural and peri-urban land into developable areas profitable. In this context, value was produced primarily through the expansion of the built-up area. Differential rent becomes more important in phases of urban maturity, demographic stagnation, and slowing construction growth. Under these conditions, valorization shifts from extending the city to transforming what already exists: building rehabilitation, functional conversion, the heritagization of the historic built environment, and touristification become the principal sources of rent extraction.

This distinction also has a historical dimension that is particularly relevant to the analysis of intermediate cities. Marginal and differential rent are not simply two alternative modes of valorization. Rather, they tend to follow one another cumulatively. Each cycle of valorization creates the conditions for the next through the relative devaluation of spaces that were previously central. Peripheral expansion fuelled by marginal rent has often contributed to the depopulation of historic centres and the reduction of their use value; this process has in turn created the conditions for rent gaps that now make differential valorization through touristification and functional conversion profitable. What appears to be a contemporary transformation is therefore the outcome of a longer trajectory in which different forms of rent reinforce one another.

This perspective refers back to a theoretical tradition that paid particular attention to the role of rent in Italian urban development. From the 1960s onwards, part of Italian critical planning and sociology interpreted urban growth as a process structurally organized around the extraction of land and property rent (Astengo, 1966; Campos Venuti, 1967; Cederna, 1956; De Lucia, 1989; Parlato, 1970; Salzano, 1998; Berdini, 2008; Tocci, 2009; Blečić, 2017). Now marginal in international debate, it is especially useful for interpreting Southern European intermediate cities, where rent remains central despite the absence of the advanced financialization found in global metropolises.

Comparative scholarship on European housing regimes has shown that Italy, Spain, Portugal and Greece share several structural features: high rates of home ownership, a limited social-housing sector, the central role of the family in reproducing housing conditions, and the widespread assetization of housing (Allen et al., 2004; Arbaci, 2019; Di Feliciantonio and Aalbers, 2018). Rent there tends to be less concentrated among large financial operators and more widely distributed among a multitude of small property owners – which does not diminish its importance but broadens its social and political base.

It is on this terrain that Parlato’s (1970) concept of the real-estate bloc (*blocco edilizio*) retains considerable explanatory power. The term refers not to a coalition of organized economic interests but to a broader social formation bringing together developers, sectoral professionals, local administrators, investors and small property owners around the defence of property valorization. The distinctive feature of the Italian case is the diffuse and interclass character of this coalition: home ownership functions simultaneously as a safe-haven asset, a means of wealth accumulation and a mechanism of social security, building broad consent around the protection of rent.

Recent international scholarship has approached the politics of property valorization through the growth machine (Logan and Molotch, 1987) and the real estate state (Stein, 2019). Both, as we argue in Section 6, presuppose a relatively clear separation between organized rent interests and the resident population – a separation that the notion of the real-estate bloc precisely denies.

From this perspective, rent is not merely an economic phenomenon but a political-institutional configuration that steers urban transformation and conditions the ability of public authorities to regulate it. The difficulties encountered by attempts at the public capture of land value uplift, the weakness of housing policies and the limited regulation of touristification are not simply technical problems: they reflect a social and political formation for which rent is a principal mechanism of reproduction.

The trajectories of transformation observed in the intermediate cities analysed in the following sections can be interpreted in the light of this theoretical framework.

## 3 Cases and methodology

### 3.1 Case selection and comparative design

The category of the non-competitive intermediate city is stipulative, and it is worth saying what it is stipulated for. It follows from the critique of competitivity developed in the grounded city argument (Engelen et al., 2017), which contests the orthodoxy – dominant in urban economics and in the policy discourse it has shaped – that treats the city as an engine of agglomeration and growth, to be assessed by its success in attracting mobile investment and talent. Against it, that argument proposes judging cities by whether they supply the foundational goods and services on which residents’ everyday welfare depends, and treating the unearned increment in land value as an object of public capture rather than as a measure of urban success. Both halves bear directly on this study: the first supplies its question – how well do cities sustain the ordinary life of the people who live in them? – and the second identifies rent as the mechanism through which that capacity is eroded.

Assessed in this way, the places that matter analytically are no longer only the competitive ones. The question has become pressing precisely as growth has ceased to be a plausible horizon for much of the European urban system and, in many of these places, ceased to be a desirable one, while liveability has become urgent. This is what moves the object of analysis away from the large and the competitive, towards cities that are neither, and which we expected to display liveability characteristics of their own – an expectation the research has borne out. The criteria that follow are derived from that purpose rather than from any pre-existing typology.

Four conditions define the class, and they come in two pairs. The first pair establishes non-competitiveness: a population of roughly 80,000 to 300,000 together with regional or sub-regional centrality – these cities organize the administration, services and labour market of a wider territory without belonging to the national or European hierarchy of competitive metropolises – and a productive base lacking an export-oriented specialization capable of sustaining endogenous growth, so that public employment, education, health and care, retail and, increasingly, tourism constitute the core of the local economy. The second pair establishes why rent, rather than gentrification, is the pertinent lens through which to examine their liveability: a housing system of the Southern European type, in which homeownership is very high, the social-housing sector residual, housing assets are transmitted within families and institutional landlords are largely absent (Allen et al., 2004; Aalbers, 2016; Arbaci, 2019; Di Feliciantonio and Aalbers, 2018); and a historic centre of recognized heritage value that lost resident population during the second half of the twentieth century and has since become the object of upgrading and functional conversion.

Applied to the Italian urban system, these criteria identify a substantial and well-defined population of cities, which the project has mapped statistically. It is from within that population that the three cases examined here were selected.

Cagliari, the capital of Sardinia (approximately 149,000 inhabitants at the 2021 census), Catania, Sicily’s second-largest urban centre (approximately 295,000), and Lecce, the capital of the Salento area in Apulia (approximately 93,000), satisfy all four conditions. They were chosen, however, not because they are similar, but because they hold these conditions constant while varying substantially along the dimensions that plausibly modulate their effects: size, and therefore the thickness of the local property market; morphological setting, from the environmental and military easements that channelled growth in Cagliari to the unconstrained mainland expansion of Lecce; institutional status, Catania a metropolitan city under the 2014 national legislation and Cagliari under Sardinian regional law 2/2016 and Lecce a provincial capital; the timing and governance of tourism; and planning trajectory, from Lecce’s early URBAN-funded rehabilitation of its historic centre to Catania’s long history of weak regulation and informal building practice. Selecting on a shared structural condition while maximizing variation on modulating factors is what allows us to distinguish the mechanism from its local expressions: if the same cumulative sequence of rent extraction is observable in settings this different, its operation cannot be attributed to any one of the features that separate them.

This is a comparison in the relational rather than the variable-based sense (Robinson, 2011, 2016; Ward, 2010; Hart, 2018). We treat the three cities not as units on which to measure the net effect of a factor, nor as points along a single developmental scale, but as three instantiations of a common regime of valorization whose divergent outcomes are themselves informative. The comparative work therefore consists in identifying recurrent configurations rather than in ranking the cases by degree of transformation, which is why we resist reading them as successive stages of one process: as Section 4 shows, the two axes along which they vary do not coincide.

Southern Italy is a deliberate starting point, on three grounds. It presents the Southern European housing regime in a particularly legible form, so that studying the diffuse and interclass structure of rent where it is most pronounced makes visible a configuration that exists, in attenuated form, across the Northern Mediterranean. It is the context in which the theoretical tradition we seek to return to international circulation was formulated. And, as the analysis that follows shows, its intermediate cities exhibit profiles of their own: a productive base with few alternatives to tourism, a property market almost untouched by institutional investors, historic centres emptied early by peripheral expansion and refilled by uses rather than by residents, and – the finding we consider most distinctive – a valorization that proceeds by consent rather than by conflict. These are not attenuated versions of metropolitan patterns but a configuration in its own right, and it is that specificity, rather than convenience of access, which warrants treating Southern intermediate cities as an object of study rather than as a residual category.

The three cities are not a statistical sample. What we offer is analytical generalization to the class defined above (Flyvbjerg, 2006; Yin, 2014), which includes a substantial share of the urban systems of Southern Italy, Spain, Portugal and Greece. What travels is the analytical framework – the cumulative articulation of marginal and differential rent, the closure of the rent gap through use rather than resident replacement, and the interclass composition of the real-estate bloc – and not the specific configurations we observe, which depend on local morphology, planning history and tourism trajectory. It matters equally to state where we expect the framework not to hold, since a proposition that accommodates every case explains none; we set out those boundary conditions in Section 7.

### 3.2 Data and methods

The study adopts a convergent mixed-methods design drawing on four bodies of evidence. The three city reports on which this article draws – containing the full data series, the interview corpus and the affordability calculations – are publicly available in Italian (GC-LIFES, 2025).

#### 3.2.1 Secondary quantitative data

Demographic, socio-economic and housing data from the ISTAT censuses of 1991, 2001, 2011 and 2021, together with tourism statistics, were reprocessed at municipal and sub-municipal level, using each city’s official neighbourhood partition, in order to reconstruct long-term trajectories and intra-urban differentiation. Property values and rents are drawn from the Real Estate Market Observatory (Osservatorio del Mercato Immobiliare, OMI) of the Italian Revenue Agency for 2004–2023 at the level of the OMI microzone, with zones reclassified after 2014 harmonized to preserve the time series. Because zoning granularity differs across the three cities, values are interpreted as within-city trajectories rather than as cross-city levels. Affordability follows the protocol of the Osservatorio Casa Abbordabile (OCA - Osservatorio Casa Abbordabile, 2023): the floor area purchasable or rentable without exceeding 30 per cent of income, computed for two INPS occupational categories on full-time permanent contracts, using provincial average incomes from INPS and MEF regional series, and with OMI values adjusted against asking prices from property platforms; the full procedure is set out in GC-LIFES (2025).

#### 3.2.2 Survey

A survey on urban liveability was administered to 1,514 residents – 504 in Cagliari, 495 in Lecce and 515 in Catania – covering perceptions of housing costs and accessibility, the availability of essential services, the effects of tourist pressure, and attitudes towards the regulation of short-term rentals. Unlike the secondary data it provides primary evidence produced by the research.

#### 3.2.3 Qualitative fieldwork

We conducted 57 semi-structured interviews across the three cities between November 2024 and February 2025: 20 in Lecce, 19 in Catania and 18 in Cagliari. Participants were selected purposively, to cover three analytically defined positions in relation to urban rent rather than to be demographically representative: long-term residents of historic neighbourhoods, including both people born there and people who moved in during the period of tourism-led valorization; shopkeepers, restaurateurs and accommodation operators, from businesses established in the 1970s to recent openings; and institutional and collective actors, among them municipal government, the regional public-housing agency, the Revenue Agency’s Real Estate Market Observatory, estate agencies, planning academics, trade associations, a tenants’ union and neighbourhood organizations. Interviewing was complemented by direct observation of the uses and rhythms of public space at different times of day and week, and by informal conversations recorded in fieldnotes.

The interview guide was organized around the analytical categories of the study: the accessibility of essential services and social infrastructure, the cost of living in the neighbourhood, the relationship with tourist flows and accommodation supply, and the coexistence of residential use with the night-time and recreational economy. All interviews were transcribed verbatim and coded thematically, combining a frame derived from those categories with codes generated from the material itself, of which the two most consequential concerned the perceived inevitability of tourism-led valorization and the loss of control over neighbourhood space.

#### 3.2.4 Documentary sources

Master plans, municipal urban plans and detailed plans for the three historic centres were analysed to reconstruct the role of policy instruments in the production and governance of rent – both what they regulate and what they leave unregulated.

#### 3.2.5 Integrating the strands

The four bodies of evidence were analysed separately and integrated at the interpretive stage, following a convergent parallel logic. Each answers a different question: the secondary data establish whether and where rent gaps opened and closed; the survey, how widely the resulting pressures are perceived and how they are evaluated; the interviews and observation, through which practices and meanings the process is enacted and accepted; the documentary analysis, what made it institutionally possible. Integration was organized through a joint display crossing the three cities with the three pillars of foundational liveability, each cell assembling the quantitative indicators, survey items and interview material bearing on that dimension.

Discrepancies between strands were treated as analytically productive rather than as measurement error, and two of them shaped the argument decisively. The first is the Lecce anomaly: census data show the historic centre gaining population, households and resident-occupied dwellings, while OMI rents, platform listings, the composition of commercial activity and the interview material all indicate an erosion of the residential function. Neither strand alone is interpretable; together they yield the article’s central finding, that the rent gap closes through the replacement of uses rather than of residents. The second is the gap between the intensity with which property pressure is perceived and the weakness of demand for its regulation, documented in Section 4.4: the survey establishes that the pattern exists and is consistent across the three cities, while the interviews supply the explanation. In both cases the quantitative strand identified the puzzle and the qualitative strand resolved it; neither is subordinate to the other.

## 4 Results: forms of rent in the intermediate city

### 4.1 Marginal rent and urban expansion in intermediate cities

A first dimension through which to observe the dynamics of urban rent in intermediate cities is marginal rent, historically associated with urban expansion and the conversion of agricultural or peri-urban land into developable areas. In all three cities this was a structuring component of twentieth-century urban trajectories, and it continues to produce significant effects in the more recent period, in selective and territorially differentiated forms.

During the twentieth-century urbanization cycle, urban expansion was neither random nor homogeneous: it was guided by planning decisions – often decisions not to act – themselves shaped by institutional arrangements, local power relations between public authorities and property operators, and morphological and environmental constraints. In all three cities expansion took place within a compromise-based rent regime typical of the Fordist period, in which the extraction of land and property rent was accompanied by relatively robust instruments of decommodification, particularly public and subsidized housing, which helped maintain broadly acceptable levels of affordability until the 1980s despite substantial growth in the built-up area.

Within this framework, a significant share of urban expansion was delivered through public-housing programmes. Only in a limited number of cases – such as the INA-Casa programme and the Piani per l’Edilizia Economica e Popolare (PEEP), the main Italian post-war public-housing programmes – did these interventions take the form of coherent projects conceived as ‘cities within the city’. More often, expansion produced predominantly residential neighbourhoods with few services, effectively creating dormitory districts. In some cases public-housing estates (*edilizia residenziale pubblica*, ERP – the Italian social-housing sector, historically residual compared with Northern European countries) were built beyond the edge of the consolidated fabric; by providing basic infrastructure to intervening areas subsequently developed by private actors, they paved the way for the extraction of marginal rent (Saiu, 2018).

The concrete forms of this process differ substantially across the three cities. In Cagliari, urban expansion in the second half of the twentieth century was shaped by the morphology of the territory and by environmental and military constraints, which channelled growth along particular axes. From the 1960s and 1970s onwards, the construction of public and low-income housing districts at the edge of the built-up area or in formerly rural zones accompanied a phase of sustained population growth while contributing to settlement dispersal. Over the long term this produced a marked divergence between an expanding wider urban system – Cagliari’s built-up area gradually merging with neighbouring municipalities that functioned as residential reservoirs – and the demographic contraction of the central municipality, which fell from 204,237 residents in 1991 to 149,092 in 2021 (−27%) while the belt municipalities expanded correspondingly: Sestu +70.7%, Capoterra +41.4%, Uta + 37.3%, Quartucciu +34.5%, Sinnai +32.4%. Between the 1991 and 2011 censuses, construction activity was concentrated in the peripheral neighbourhoods of the Pirri municipality – Barracca Manna, Monreale and Is Campus–Is Corrias – partly through unauthorized developments subsequently regularized. Decade after decade, marginal rent was appropriated precisely through this shift from the regional capital to the dispersed built environment of the metropolitan city.

In Catania, urban expansion took a more fragmented and discontinuous form, characterized by extensive dispersed urbanization and a close interplay between private initiative, weak public regulation and informal building practices. Peripheral growth, often without adequate coordination of infrastructure and services, reinforced deep socio-spatial inequalities and shifted the residential function away from central and historic neighbourhoods. Here too expansion cannot be attributed to sustained economic development: it resulted from a combination of housing demand, expectations of property appreciation, and the selective capacity of local actors to capture land rent at the edge of the built-up area. The dynamic did not end with the twentieth-century cycle but was repurposed in commercial form: Catania now has one of the highest concentrations of shopping centres among Italian cities (460 m^2^ per 1,000 inhabitants, Confcommercio, 2024), and between 2012 and 2022 local units in construction rose from 1,447 to 1,736 and those in real-estate activities from 634 to 806. Marginal rent therefore continues to operate at the urban edge and in the hinterland, sustained by weak regulation and informal building practice.

In Lecce, a smaller city, urban expansion was less intense quantitatively but no less significant qualitatively. From the 1960s onwards, growth in the built-up area accompanied a progressive shift of residence towards new peripheral districts, consistent with a housing imaginary centred on the detached house and the suburban villa. This depopulated the historic centre at a relatively early stage, well before the onset of tourism-led valorization, establishing a trajectory in which marginal rent, by driving migration from the centre to the edges, created the conditions for the later extraction of differential rent. More recent construction confirms the peripheral logic: expansion between 2001 and 2011 concentrated in the coastal settlements and in public-housing neighbourhoods built at the margins – Santa Rosa, developed under the Fanfani Plan, and Kolbe–San Giovanni Battista, which remained a marginal dormitory district. In 1991 the ageing index clearly distinguished an ‘older’ original core (Centro, Mazzini, Santa Rosa) from ‘younger’ peripheral neighbourhoods; by 2021 it had flattened around the city average (216), indicating that the expansionary cycle that emptied the centre had run its course.

Marginal rent is therefore not merely a legacy of the past, destined to disappear with the end of the twentieth-century expansionary cycle. Comparison also shows that its variations across the three cities are not differences of intensity but of institutional and morphological configuration: constrained and axial in Cagliari, fragmented and ungoverned in Catania, dispersed across modern apartment blocks and peri-urban villas in Lecce.

What the three cases share is the long-term outcome: in each, peripheral expansion helped empty central spaces. Lecce shows the sequence most clearly. The early emptying of its historic centre, produced by expansion between the 1960s and the 1980s, was the condition of possibility for its later touristification: the rent gap now exploited by tourism operators and small investors did not pre-exist the city’s recent urban history but was produced, decade after decade, through the development of peri-urban land.

### 4.2 Differential rent and the functional conversion of historic centres

While marginal rent historically structured the peripheral expansion of intermediate cities, since the 1990s a renewed capacity to extract differential rent has become concentrated in their historic centres, based not on extending the built-up area but on transforming what already exists. In the three cities analysed, this has unfolded amid demographic stagnation, declining demand for stable residence, and the growing centrality of economies of consumption, tourism and symbolic valorization, combining urban upgrading, functional conversion and changes in the uses of space.

In Cagliari, demographic data show that the historic neighbourhoods underwent a prolonged period of depopulation between 1991 and 2011, followed in the last decade by partial stabilization or slight population growth, often associated with the settlement of foreign residents and temporary or discontinuous forms of residence. Neighbourhoods such as Marina and Stampace have recently grown, but this does not amount to a full recovery of their traditional residential function; rather, it reflects selective functional conversion associated with restaurants, accommodation, and commercial activities oriented towards tourism and leisure. Other historic neighbourhoods, such as Castello and Villanova, continue instead to show demographic fragility, population ageing, and a declining supply of long-term housing. Census data sharpen the picture: between 1991 and 2021 Castello lost 15.3% of its residents (from 1,784 to 1,511) and Villanova remained essentially stable (+2.7%), while Marina grew by 13.2% (from 2,433 to 2,755) and Stampace by 26.1% (from 5,839 to 7,362). This recovery, however, does not signal the return of a stable residential function. As early as 1991–2001, the number of dwellings in the centre increased through conversion into B&Bs and holiday homes (Marina +23%, Villanova +14%, Castello +12%); by 2021, single-person households exceeded 60% in the historic neighbourhoods (Castello 64.9%, Marina 64.8%), compared with a city average of 49%, while the municipal ageing index tripled (from 88 to 312). Where population growth occurs, it is a form of ‘new filling’, not class replacement in the strict sense. In this context, differential rent manifests itself mainly through the valorization of uses and locational advantages rather than through clear social replacement.

In Catania, the transformation of the historic centre is highly discontinuous and spatially selective. Rehabilitation projects are concentrated along specific urban axes and in highly visible micro-areas, leaving large parts of the centre in conditions of housing decay, residential precarity and social marginality; functional conversion takes the form of an unstable coexistence between new tourism-accommodation uses, fragile forms of residence and the continued presence of low-income populations, which intensifies competition among uses rather than producing widespread gentrification. Demographically, the historic centre (the first municipality) declined from 58,184 residents in 1991 to 55,566 in 2021, a limited fall of 4.5%, with an initial phase of displacement followed in the last decade by renewed growth in dwellings occupied by residents. Here too, ‘new filling’ does not restore ordinary residence: the share of residents with tertiary qualifications rises – partly because of the university – and the conversion of the *bassi*, ground-floor units in working-class buildings, into short-term rentals proliferates. In Lecce, by contrast, valorization is more systematic and strongly oriented towards the city’s symbolic and cultural dimension: the widespread conversion of the building stock to accommodation uses, the aestheticization of public space and the promotion of the city as a cultural destination have transformed a centre that had already partly lost residents in an earlier period. Unlike the other two cases, Lecce’s historic centre did not depopulate between the 1991 and 2021 censuses – population, households and resident-occupied dwellings grew more there than in peripheral neighbourhoods – a result of the early activation of renovations under the URBAN programme, which nevertheless did not curb, and indeed facilitated, the explosive growth of leisure functions. Over the same period rents in the historic centre rose by 72% (2004–2023, OMI data); bed-and-breakfast establishments increased from 92 recorded in 2005 (Izzi, 2005) to more than 2,250 short-term rental listings in January 2025, about 70% of them entire apartments; and, according to a tenants’ association, of 200 contracts signed between August and October 2024 only one concerned a dwelling rented to a family. For a worker earning an industrial wage, the floor area theoretically affordable for purchase in the historic centre rose from 36 m^2^ in 2004 to 46 m^2^ in 2023, because sale prices fell after the bubble, whereas the affordable rental area declined from 30 to 26 m^2^: renting, rather than purchasing, became increasingly inaccessible precisely in the area exposed to tourist pressure. The residential function is therefore substantively eroded even where the census population rises: the rent gap closes through use replacement, not resident replacement (Figure 1).

**Figure 1:**
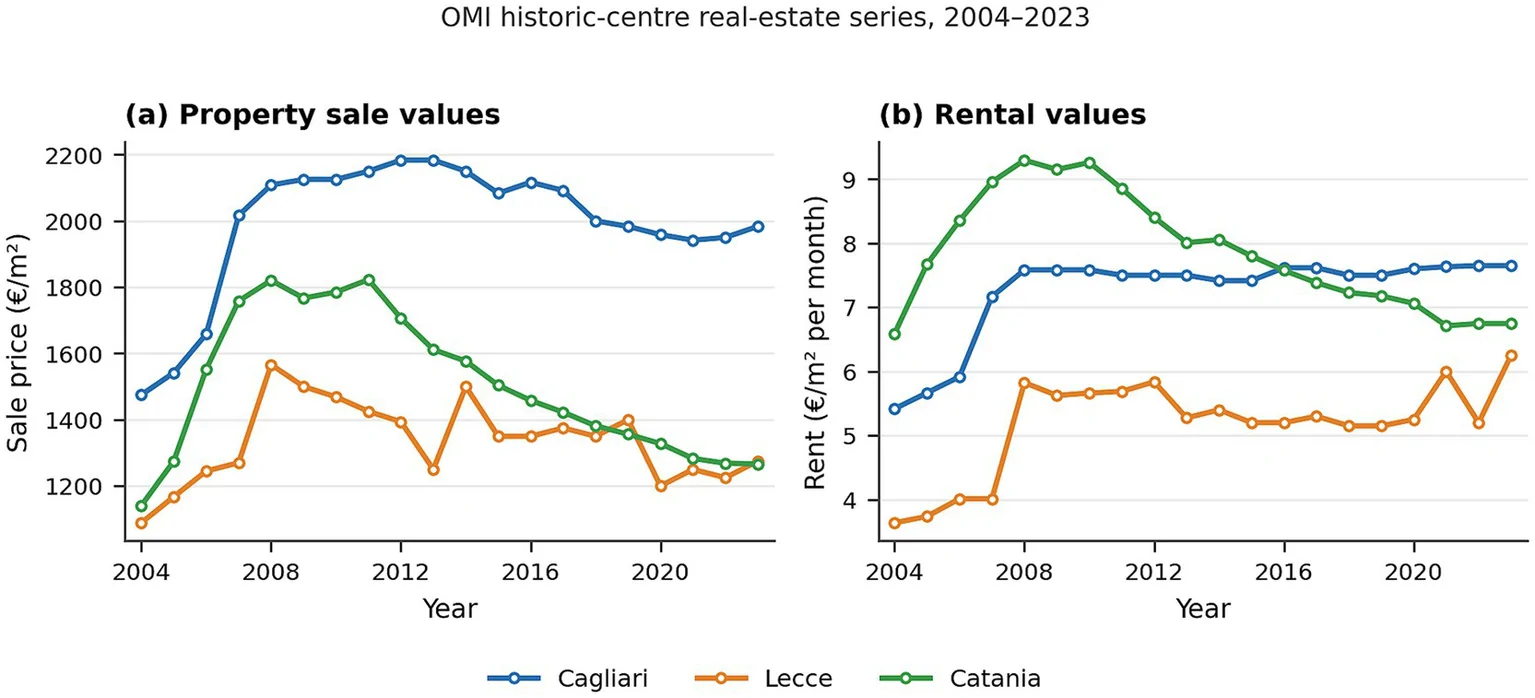
Real-estate values in the historic centres of Cagliari, Lecce, and Catania, 2004–2023. **(a)** Property sale values (€/m^2^); **(b)** rental values (€/m^2^ per month). Each line represents one city. Source: Authors’ elaboration of OMI data (Osservatorio del Mercato Immobiliare, Italian Revenue Agency). The historic-centre aggregate corresponds to the OMI “Centro Storico” zone for Lecce; the mean of central zones B1 (Marina–Stampace), B3 (Villanova), and B4 (Castello) for Cagliari; and the central-zone average for Catania. Because zoning granularity differs across cities, absolute levels are indicative and should be interpreted primarily as within-city trajectories.

In all three cases, differential rent operates through functional conversion and the redefinition of urban uses rather than through intense and generalized gentrification. Processes of social replacement in the historic centres of intermediate cities are moderate, intermittent and highly localized, involving small property owners, local investors, tourism operators and families converting part of their assets into a source of income. This fragmented character does not eliminate the cumulative effects, which emerge over the medium term through rising property values, a reduced supply of long-term housing, and increasing competition between residential and tourist-accommodation uses.

The transformation of historic centres is also closely intertwined with planning instruments. In Cagliari, the Detailed Plan for the Historic Centre provided a framework for building rehabilitation and the upgrading of public space, but had little effect on preserving the residential function. In Catania and Lecce likewise, planning has privileged formal conservation, heritage valorization and tourist attractiveness, relegating housing policies and instruments for regulating uses and property markets to a secondary position.

Comparison among the three cases identifies three distinct configurations through which differential rent is activated. Cagliari represents selective and partial valorization: the process is incomplete and spatially uneven, and gentrification remains weak and circumscribed. Catania instead represents unstable coexistence: differential rent produces sharp micro-polarizations along particular axes of accessibility and attractiveness, while leaving large areas of the historic centre in conditions of decay and marginality; the tension between new tourism-accommodation uses and fragile residence is most acute here. Lecce, finally, represents systemic and symbolic valorization: differential rent operates in a historic centre already substantially emptied of residents and is activated primarily through changing uses and the aestheticization of public space, producing a deep and relatively coherent functional reconfiguration. We return in Section 6 to what this implies for the analytical status of gentrification.

### 4.3 Touristification and rent extraction

Touristification is now one of the principal mechanisms through which differential rent is reactivated in intermediate cities. In the three cities analysed, the growth of tourist flows and related activities is not simply the expansion of an economic sector but a reorganization of urban uses that profoundly affects the residential function, the property market and the structure of economic activities in historic centres. The *mise en tourisme* is a specific mode of valorizing the existing urban fabric, consistent with a rent regime based on functional conversion and generating asymmetrical competition between residential and tourist uses.

This process creates a value differential between the residential and tourist use of housing, making it economically advantageous to withdraw dwellings from the long-term rental market (Cocola-Gant, 2016). The effects are especially visible where the historic building stock is functionally obsolete. In such contexts, high renovation costs – together with the technical and administrative burdens associated with heritage restrictions – may discourage ordinary residential use and depress use values, while expectations of higher tourism-related returns make rehabilitation investment more feasible. Tourism rent, in other words, is not merely an outcome of transformation; it becomes a lever that makes the functional conversion of the existing stock feasible and profitable.

The *mise en tourisme* also alters the composition of economic activities, reinforcing functional specialization. In all three cities food services, bars and visitor-oriented retail are expanding, accompanied by a decline in commercial diversity and in neighbourhood services for residents, so that historic centres are reoriented towards consumption and temporary use and weakened in their role as spaces of everyday life and social reproduction (Figure 2).

**Figure 2:**
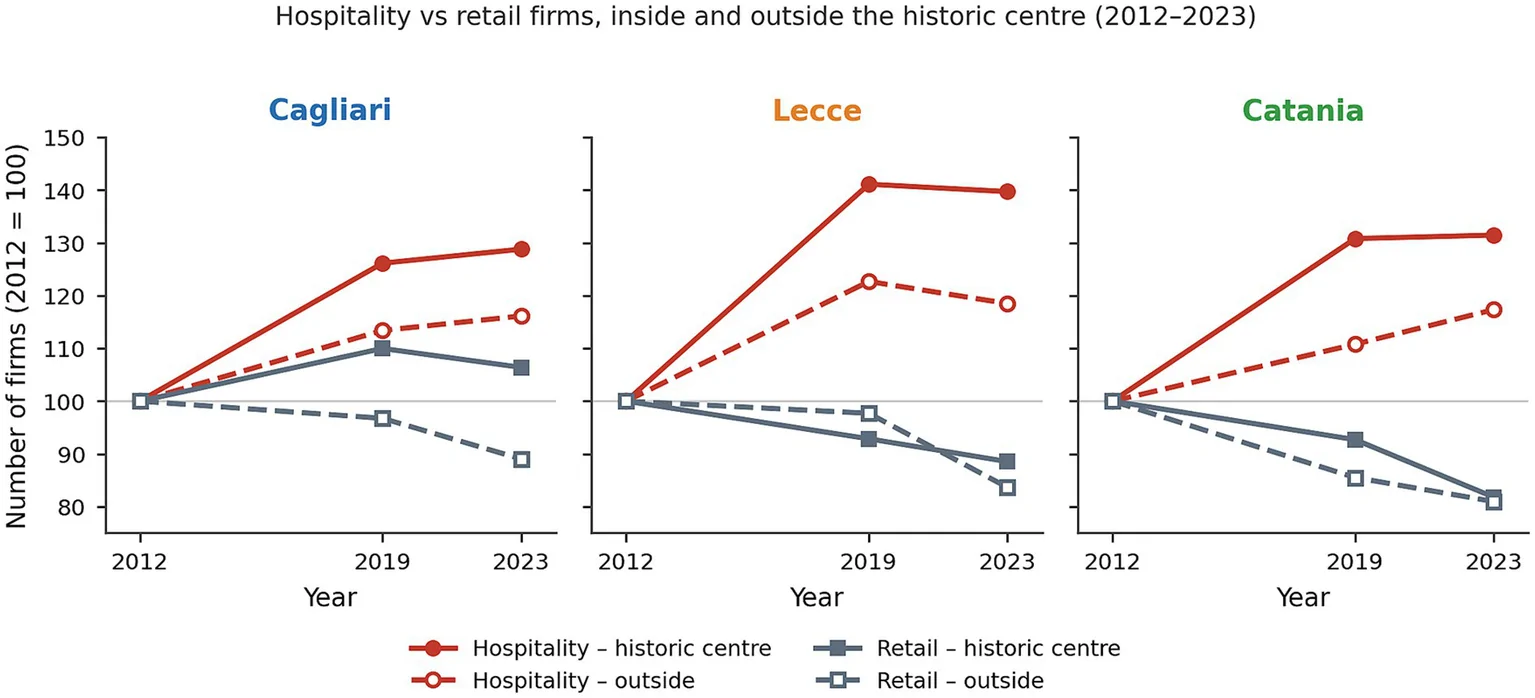
Hospitality and retail firms inside and outside the historic centre in Cagliari, Lecce, and Catania, 2012–2023 (index, 2012 = 100). Solid lines represent the historic centre; dashed lines represent the rest of the municipality. “Hospitality” includes accommodation establishments, bars, and restaurants; “retail” refers to retail trade. Data refer to three benchmark years: 2012, 2019, and 2023. Source: Authors’ elaboration of business-register data provided by Confcommercio.

A distinctive feature of intermediate cities is that touristification is not driven primarily by large global operators but rests on a multitude of micro-processes of valorization. Small property owners, families, local investors, and economic operators participate in converting properties and businesses, often in the absence of stringent public regulation. Weak instruments for governing tourism and the housing market – in both urban planning and sectoral policy – together with digital platforms facilitate this fragmentation of supply, enabling tourism rent to spread throughout the urban fabric. The phenomenon is less spectacular than in major metropolises, but highly pervasive in its effects. The scale of this fragmentation can be measured by the gap between official and ‘hidden’ accommodation supply: in Catania, the 446 establishments registered by ISTAT in 2023 corresponded to 4,413 Airbnb listings in February 2025, 74.1% of them entire apartments; in Lecce, the 1,926 establishments on the regional register were accompanied by more than 2,200 listings. Tourism rent therefore spreads in atomized form through a multitude of small property owners, largely beyond the scope of official measurement.

In Cagliari, tourist arrivals and overnight stays have grown continuously since the early 2000s, with a marked acceleration over the last decade and rapid recovery after the pandemic. Particularly significant is the more-than-proportional rise in overnight stays relative to arrivals, indicating the city’s consolidation as more than an occasional destination (Figure 3). Accommodation supply has expanded correspondingly, especially in the non-hotel sector, which draws directly on the housing stock of central neighbourhoods and intensifies competition between stable residence and temporary accommodation (Figure 4). In 2022, privately rented accommodation accounted for more than 71% of tourist establishments in Cagliari, about 40% of bed spaces and over 35% of total capacity; in 2023 the municipality accounted for 39.2% of normalized property transactions in the whole of Sardinia. The relative stability of long-term rents in central neighbourhoods since 2008 is plausibly attributable to the withdrawal of stock into short-term rentals: not a sign of easing market pressure, but the effect of the best units leaving the residential circuit.

**Figure 3:**
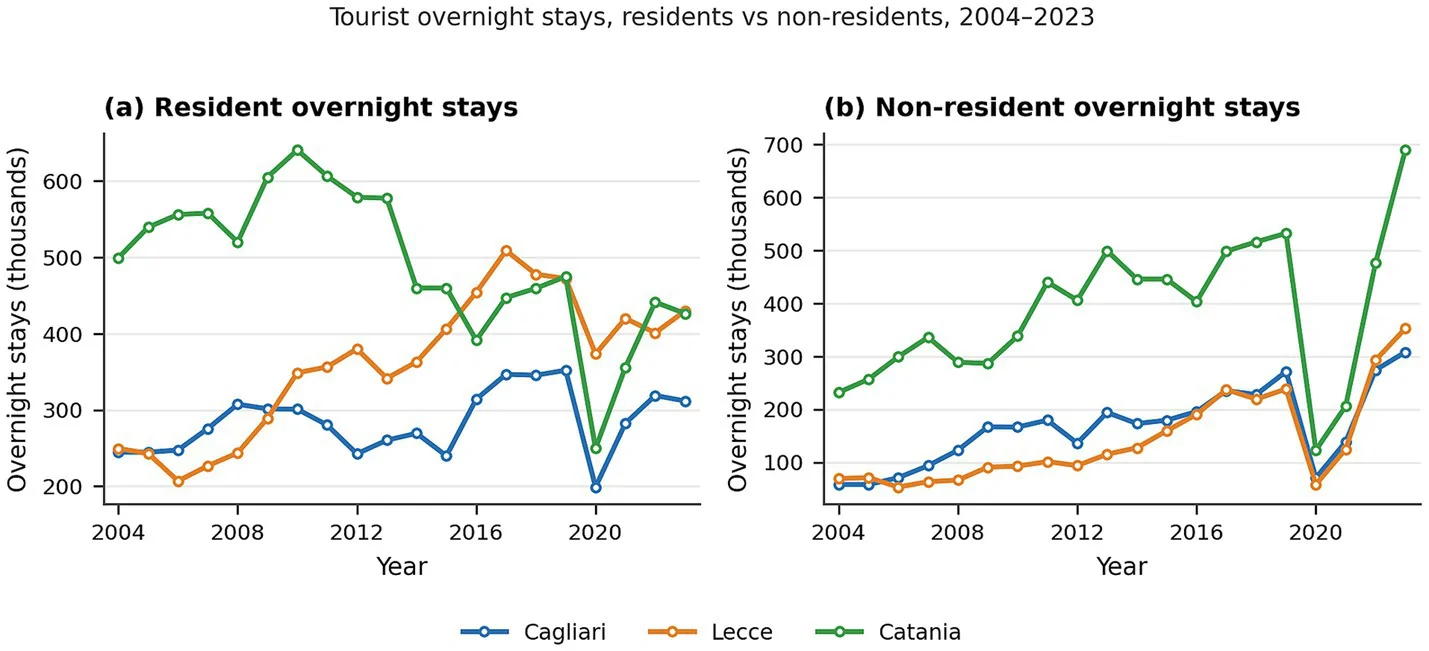
Tourist overnight stays in Cagliari, Lecce, and Catania, 2004–2023, by guest origin. **(a)** Resident guests; **(b)** Non-resident guests. Values are expressed in thousands of overnight stays and refer to all types of accommodation establishment. Source: Authors’ elaboration of ISTAT tourism statistics.

**Figure 4:**
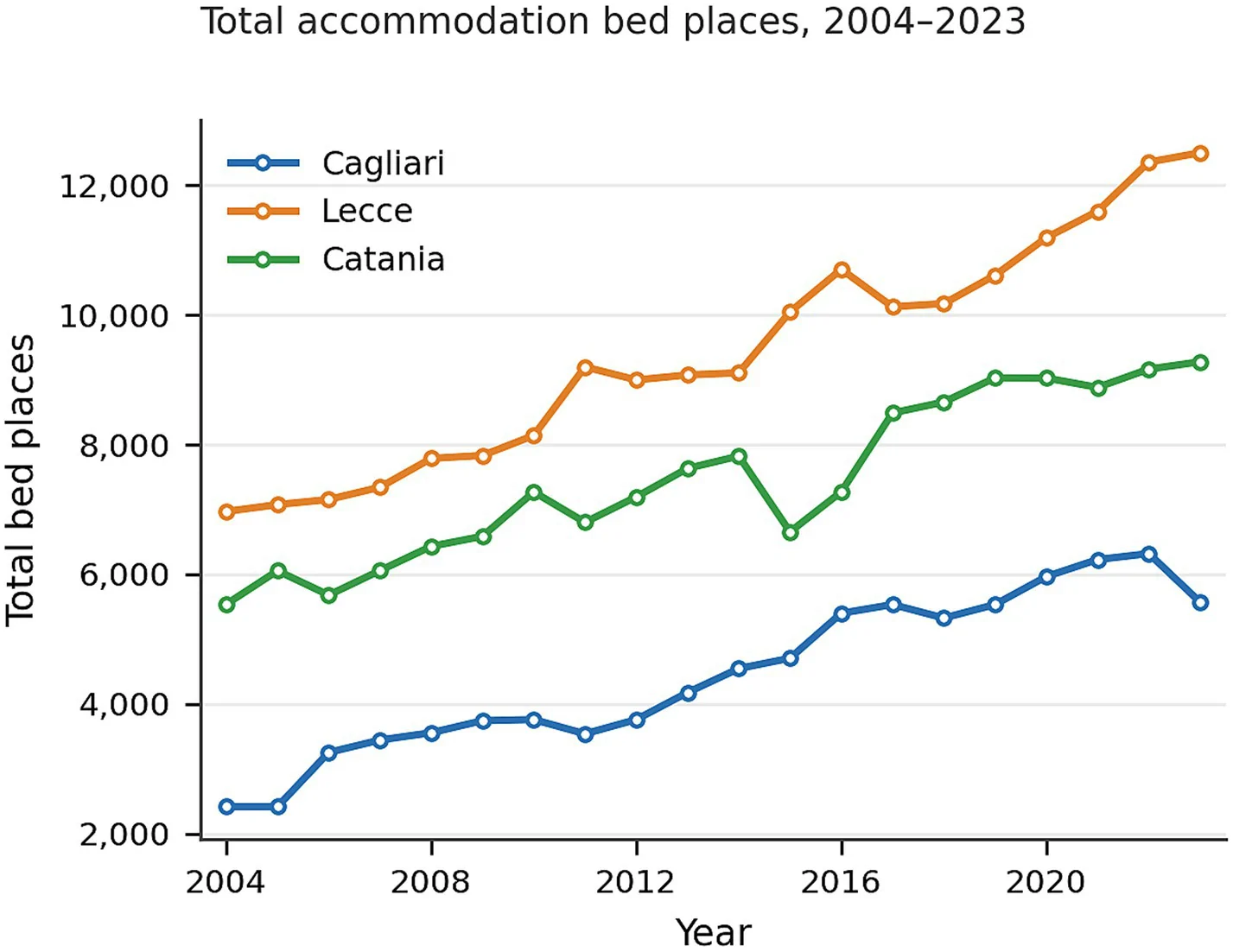
Total accommodation capacity in Cagliari, Lecce, and Catania, 2004–2023. Values refer to the total number of bed places. Source: Authors’ elaboration of ISTAT tourism statistics. ISTAT’s municipal-level breakdown by establishment type is available only up to 2012 and is therefore not shown.

In Catania, the growth of tourism is superimposed on an urban fabric marked by sharp socio-spatial inequalities, deteriorated housing and weak regulatory capacity, and is correspondingly selective, concentrating in parts of the historic centre and along particular accessibility axes. Most significantly, pressure does not derive from rising tourist flows – arrivals in 2023 returned close to, but did not exceed, 2019 levels, while official accommodation supply remained static at 446 establishments, only two more than in 2019 – but from the conversion of the existing stock. Food-service establishments increased by 28% between 2012 and 2022, from 1,259 to 1,615, while around 3,200 platform listings indicate tourist use of residential space. It is the transformation of uses, more than the growth in tourist numbers, that withdraws housing from residence. In Lecce, by contrast, touristification is systematic and symbolically central to the city’s recent trajectory. Arrivals and overnight stays more than doubled between 2004 and 2019 and rose further in 2023; short-term rental listings increased from just over 100 in 2012 to more than 2,200 in 2024; and accommodation establishments expanded much more rapidly in the historic centre (+40%) than in other neighbourhoods (+19%, Confcommercio, 2024), alongside a corresponding contraction in neighbourhood retail.

The gradient emerging from the comparison – from emerging touristification in Cagliari, through selective touristification in Catania, to systemic touristification in Lecce – does not represent stages of a single evolutionary process, nor merely differences in intensity. It reflects distinct configurations of the relationship between urban heritage, the tourism market and local governing capacity, which produce different risk profiles for foundational liveability: the erosion of residual income in Cagliari, spatial polarization in Catania, and in Lecce a further contraction of functional diversity and of the supply of long-term housing, including outside the historic centre.

### 4.4 Residents’ perceptions: the consensual closure of the rent gap

The data presented thus far document the structure of rent and its morphological transformations. The survey described in Section 3.2 adds the dimension of perception. It reveals what we consider one of the study’s most significant findings: in Southern Italian intermediate cities, the rent gap closes without generating social conflict because residents perceive the tourism-led valorization of urban space not as a threat but as an opportunity, even where property pressure is experienced most intensely.

Perceptions of property pressure are extremely high and widespread. Asked whether rental and property prices had risen in their area, 86.3% of residents in Cagliari, 93.5% in Lecce and 86.7% in Catania responded affirmatively; an increase in short-term rentals in their own neighbourhood was perceived by 74.8, 84.0 and 66.4%, respectively. Residents clearly see the transformation of the property market and recognize its connection with touristification, and they experience it directly: 57.8% of respondents in Cagliari, 60.8% in Lecce and 49.7% in Catania had encountered difficulty finding a rental dwelling. Housing costs weigh particularly heavily on income in Lecce, where 24.3% of households spend more than 30% of their income on housing and 44.7% between 20 and 30%.

Despite this awareness, attitudes towards tourism remain clearly positive. Asked to assess whether the presence of tourists ‘increases the vitality and opportunities of the local area’ on a scale from 1 to 5, residents expressed high and broadly uniform agreement: 4.03 in Cagliari, 4.11 in Lecce, 4.10 in Catania. The opposite item – tourism ‘causes problems of coexistence or disturbance for residents’ – remained below the neutral point in all cases (2.30, 2.62 and 2.48 respectively). Lecce is especially telling: it is the city where property pressure is perceived most intensely, yet the assessment of the ‘vitality’ generated by tourism far exceeds that of the ‘disturbance’ it causes. Perception of pressure and positive evaluation of tourism coexist within the same population.

Even more significantly, awareness of property pressure is not accompanied by a demand for regulation. Average agreement with the statement ‘my neighbourhood needs more rules to limit short-term tourist rentals’ fell below the neutral point in Cagliari (2.30) and Catania (2.06), and remained below it in Lecce (2.79). Yet residents openly recognize the transformation under way, assigning high scores to the statement that ‘over the last 5 years, the city has become more tourist-oriented’ (3.75 in Cagliari, 4.02 in Lecce, 3.63 in Catania). They see the city changing under the pressure of tourism-led valorization, yet most do not demand that the change be restricted.

The coexistence of these three elements – high perception of pressure, a positive assessment of tourism, and weak demand for regulation – cannot be read as apathy or lack of awareness. Its explanation is structural: in urban economies with few productive alternatives, the tourism-led valorization of rent is perceived as the only available source of opportunity and therefore as essentially inescapable. Tourism as ‘vitality and opportunity’ is the other side of the absence of economic alternatives.

In this sense, we may speak of the consensual closure of the rent gap. Whereas in metropolises touristification and property valorization generate – albeit in scattered forms – urban conflict, right-to-housing movements, and explicit demands for the regulation of short-term rentals, in Southern intermediate cities the same process unfolds without significant friction. The differential between potential and capitalized rent closes not because residents fail to perceive its effects, but because those effects are accepted as the price – or even the sign – of an economic vitality otherwise out of reach. This is both one reason why rent is valorized so efficiently in these contexts and one of the principal obstacles that any regulatory policy will encounter. We return to this issue in the discussion.

## 5 Effects on foundational liveability: residence, affordability, and everyday life

The dynamics of marginal rent, differential rent and touristification analysed in the preceding sections have significant cumulative effects on the residential function and on the foundational liveability of intermediate cities. The term ‘liveability’ is used here critically, and deliberately distanced from its dominant meaning in urban policy discourse, which has turned it into a normative and competitive category – an index of attractiveness measured through international rankings and mobilized to promote cities in global markets for investment and talent (Wetzstein, 2016). Understood in that way, liveability is identified with the desirability of urban space for the middle classes, visitors and investors, concealing the fundamental question: liveability for whom? (Marcuse, 2009).

An alternative perspective is provided by the concept of foundational liveability developed within the foundational economy approach (Foundational Economy Collective, 2018; Froud et al., 2018; Calafati et al., 2023). Liveability here is not a competitive quality of urban space but a material condition of everyday life, structured around three interdependent pillars: households’ residual income, once essential housing and transport costs have been met; the availability and accessibility of essential services across the territory; and the quality of social infrastructure, understood as the spaces, relationships and practices that enable the reproduction of collective life. It cannot be captured by aggregate indices and must be analysed in disaggregated terms, since a city may be fully liveable for some sections of its population and structurally unliveable for others (Froud et al., 2018). In non-competitive cities, where disposable incomes are lower and skilled employment scarcer, every increase in housing costs produced by property and tourism-led valorization directly reduces residual income (Bricocoli and Salento, 2020). This perspective intersects with that of the grounded city (Engelen et al., 2017), which places neighbourhood economies and the everyday life of permanent residents, rather than the attraction of capital and visitors, at the centre.

In the cities analysed, the effects on foundational liveability do not primarily take the form of rapid, large-scale displacement. They emerge instead through slow and often inconspicuous transformations that progressively affect all three pillars: housing costs erode residual income; touristification and commercial specialization reduce the availability of neighbourhood services; and changes in the rhythms and uses of urban space degrade the social infrastructure of everyday life. This is the silent and cumulative erosion that Cocola-Gant (2023) calls place-based displacement: not so much the loss of a home as the loss of place – the network of services, relationships and everyday practices on which the experience of dwelling had rested.

### 5.1 Who lives there: demographic structure and residential composition

A first indicator of foundational liveability concerns the demographic and household structure of central neighbourhoods, which across the three cases shows convergent signs of qualitative transformation in residence even where the quantitative data show no net population loss. Cagliari illustrates the pattern: pronounced ageing, single-person households approaching half of all households in the municipality and exceeding 60 per cent in some historic neighbourhoods, and a sharp decline in the presence of children, indicating reduced attractiveness for families and a structural difficulty in sustaining stable residence. Ageing and household fragmentation recur in Catania, compounded by persistent socio-housing fragility and precarious or transient forms of residence, and in Lecce, where a demographically more vibrant centre is associated with more selective socio-economic profiles and fewer large households. In all three, the supply of housing for stable residence contracts even where the census population does not, as dwellings pass into short-term rental or intermittent occupation.

### 5.2 What it costs: housing affordability and residual income

These dynamics directly affect the first pillar of foundational liveability: households’ residual income. Intermediate cities have lower average price levels than major metropolitan areas, but rising housing costs weigh heavily on local incomes, which are less dynamic and structurally lower. Differential rent and touristification therefore produce a ‘relative’ housing-cost crisis that is particularly burdensome precisely because the relationship between prices and incomes is intrinsically less favourable.

In Cagliari, data from the Real Estate Market Observatory show that, while purchase prices declined after the 2008 financial crisis, rents followed the opposite trajectory, rising gradually but continuously throughout 2004–2023. Applying the conventional affordability threshold of 30% of income, calculations based on OMI and INPS data show that in 2004 a permanent employee could theoretically afford to rent 32–35 m^2^ in the historic centre; by 2013, that area had fallen to 21–24 m^2^; and in 2023, despite a partial recovery, it remained around 26–30 m^2^. The gap between housing costs and local spending capacity is widening precisely in the historic neighbourhoods affected by touristification.

In Catania, competition between residential and tourist uses reduces the supply of long-term rentals in the most attractive central areas, constraining housing access in ways that disproportionately affect low- and lower-middle-income groups, including migrant workers who are often excluded from the formal rental market. In Lecce, where touristification is most advanced, the rent increases and the concentration of platform listings among multi-listing hosts documented in Sections 4.2 and 4.3 translate directly into a contraction of the ordinary residential market. Housing is thus becoming progressively less accessible precisely where services, infrastructure and urban quality are concentrated, and in the absence of the strong demographic pressure that might otherwise explain the trend. This documents the mechanism the article identifies as specific to non-competitive ordinary cities: rent erodes foundational liveability not through the direct displacement of existing residents, but through the progressive exclusion of those who would like to move in and of those who can no longer afford to remain – a form of displacement that does not entail gentrification in the strict sense, but is clearly visible in affordability data and in the demographic composition of central neighbourhoods (Arbaci et al., 2021).

### 5.3 What is missing: essential services and the de-residentialization of urban space

The transformation of the commercial and service fabric affects the second pillar of foundational liveability. Data on local units and employment show a substantial strengthening of accommodation and food services in the central neighbourhoods of all three cities, accompanied by a relative contraction of the neighbourhood businesses – food shops, pharmacies, personal services – that constitute the everyday infrastructure of residential life. The consequences fall unevenly: they are borne most heavily by older residents and by those with limited mobility, for whom the disappearance of a nearby food shop is not an inconvenience but a constraint on the possibility of continuing to live there.

This functional specialization amounts to a de-residentialization of central urban space: the centre progressively loses the functions of everyday social reproduction and assumes those of temporary use, becoming more dependent on temporary users and more costly to inhabit for permanent residents – particularly older people and those with limited mobility, who are progressively leaving touristified historic centres.

### 5.4 How people live: coexistence conflicts and social infrastructure

These processes affect the third pillar of foundational liveability: social infrastructure. Coexistence between residents and tourist flows generates tensions around noise, crowding and the intensive use of public space during evening and night-time hours, as the night-time economy organizes the city’s timetable around visitors’ needs rather than residents’, and the research interviews document these conflicts with remarkable consistency across the three contexts. A recurrent theme in the testimonies collected is the perceived loss of control over neighbourhood space: once the tourist presence exceeds a certain threshold, not only tranquillity but the very possibility of sustaining one’s decision to live in that area is undermined. This is what Cocola-Gant (2023) means by place-based displacement – the gradual disintegration of the places of everyday life and the loss of the reference points and neighbourhood relationships on which dwelling had rested – and what Fullilove (2016) calls root shock. The absence of displacement does not mean that residents have not lost the conditions that make residence possible.

At the same time, rising rents and changes in property use fuel conflict between residents and owner-investors, gradually redefining power relations in urban space, and in all three cases this is compounded by conflict with local authorities perceived as unable to govern the cumulative effects of tourism-led and property valorization. Absent adequate regulatory instruments, such tensions are managed in fragmented or emergency-driven ways, reinforcing feelings of marginalization and loss of belonging to one’s places of everyday life.

## 6 Discussion: urban rent as an interpretive key to foundational liveability and the development trajectories of intermediate cities

Non-competitive intermediate cities are neither marginal spaces nor attenuated versions of the metropolis. It is in these contexts that the central argument of this article is most clearly demonstrated: gentrification is a specific and contingent outcome of urban rent, not its paradigm. The comparative analysis of Cagliari, Catania and Lecce shows that rent extraction has profound and systematic effects on foundational liveability – the compression of residual income, the depletion of essential services and the destabilization of social infrastructure – regardless of whether residents are replaced. The relevant question is therefore not whether and to what extent cities display signs of gentrification, but how rent restructures the uses of urban space and the material conditions of dwelling, even in its absence.

The idea that the most critical processes of valorization and de-residentialization are the preserve of major metropolises must be abandoned. In Cagliari, Catania and Lecce, touristification, the erosion of residential functions and the crisis of neighbourhood services unfold in contexts of shallower property markets, lower average incomes and weaker regulatory capacity, which is precisely why their effects on foundational liveability are often more destabilizing than in metropolises, where greater scope for public compensation exists. Rent here is driven not by global capital flows but by a plurality of local actors working within a fragmented regulatory framework – a capillary structure that makes transformation less visible and less spatially concentrated, but no less pervasive.

The relationship between marginal and differential rent should be understood as an interdependent and cumulative process, not as a succession of phases. The sequence has a quasi-dialectical structure in which each cycle of valorization produces the conditions for the next through the devaluation of spaces valorized in the preceding one. By emptying historic centres, the peripheral expansion of the second half of the twentieth century constructed them as spaces of opportunity, accumulating a widening gap between their residual use value and the exchange value later extracted through functional conversion. No conscious agent orchestrated this. It was the aggregate outcome of a multitude of decisions by families, developers and investors who, in each phase, followed the dominant housing imaginary – first the modern apartment block with a lift, then the detached house with a garden, and finally the restored historic residence – each of which signalled upward mobility relative to the preceding cycle and rendered what had previously been valorized obsolete. A historic neighbourhood thus empties not because anyone planned it, but because development pressures and administrative choices encouraged a different form of expansion; it is later rediscovered – first by young people, then by tourists, and finally by investors – both because public authorities adopt ‘upgrading’ measures and because the differential between residual use value and valorization potential has reached the threshold of profitability for the operators available: small property owners, accommodation providers and restaurateurs. When gentrification appears, it is the terminal phase of this process, not its engine, and in non-competitive ordinary cities that phase often does not arrive at all. What remains instead is the silent erosion of foundational liveability produced by all the preceding cycles combined.

Foundational liveability is the central stake in this process and must be conceptualized in a radically different way from its use in competitive urban policies. It is not an index of attractiveness or a quality measurable through international rankings; it is a material condition, as conceptualized in Section 5, systematically eroded by rent dynamics in non-competitive intermediate cities (Froud et al., 2018; Calafati et al., 2023; Engelen et al., 2017). The cities examined invest in liveability as a factor of external attractiveness, but in doing so undermine its material foundations for permanent residents. This paradox is the specific form assumed by the conflict between the use value and exchange value of housing in non-competitive ordinary cities (Madden and Marcuse, 2016): not a clash between gentrifiers and displaced residents, but the progressive and capillary erosion of the conditions that make it possible to inhabit central places.

The configurations identified in Sections 4.2 and 4.3 do not line up. The axis of differential rent and the axis of touristification order the three cities differently, which is why they cannot be read as successive stages of a single evolutionary process. What the cases share is not a position on a scale but a structure: a cumulative sequence in which marginal rent created the conditions for differential rent, which created the conditions for touristification, which erodes foundational liveability the more severely the fewer alternatives the local economic structure provides.

The diffuse and capillary structure of rent in Southern intermediate cities cannot therefore be explained solely by weak planning instruments or the fragmentation of the property market. It is the product of a political formation that the Italian critical tradition identified with precision half a century ago, but which the principal Anglo-American theories of urban capitalism fail to capture. Logan and Molotch’s (1987) growth machine describes a coalition of local elites maximizing exchange values against use values, and Stein’s (2019) real estate state the penetration of property capital into state structures; both presuppose a relatively clear separation between organized rent interests and the resident population. The real-estate bloc (Parlato, 1970), by contrast, shows that in urban economies based on widespread property ownership the defence of rent is an interclass interest that permeates the social fabric and is reproduced through ordinary forms of political consent. This is not a homogeneous social body but a varied composition ranging from speculators, who occupy a hegemonic position, to small property owners; it is nevertheless mobilized instrumentally against any attempt to tax or publicly capture rent. According to a populist commonplace that appears resistant to disproof, any such measure affects very broad sections of the property-owning population – which helps explain why reforms are systematically neutralized by socially extensive coalitions. The configuration is particularly deeply rooted in Southern intermediate cities, where the continuity between the construction cycle, planning and local political power has been widely documented (Cederna, 1956; Sacco, 1982). The limited capacity of local institutions to govern the effects of rent is therefore constrained not only by contingent factors – inadequate instruments, insufficient technical staff, limited financial resources – but by a systemic configuration linked to the persistence of the real-estate bloc as a political formation opposed to reform.

Urban policies for intermediate cities cannot therefore be limited to importing instruments designed for different contexts. The regulation of short-term rentals, restrictions on tourist lettings and plans to protect stable residence exist in various forms in major European cities, where they were conceived as responses to gentrification and overtourism. Where the real-estate bloc is more capillary and rent has a broader social base, the same instruments risk being politically neutralized before they are implemented, because they affect interests held not only by elites but by broad sections of the property-owning population.

The lesson of the Italian critical tradition is relevant here: the problem of rent cannot be addressed without constructing a socio-political bloc alternative to the real-estate bloc. This means identifying those with an objective interest in regulating rent – tenants, workers in neighbourhood services, shopkeepers displaced by tourism specialization, residents exposed to coexistence conflicts, young families excluded from affordable housing – and equipping them with instruments of representation and bargaining power. Within this framework urban plans are not merely technical instruments but political devices that can either neutralize or enable such a coalition. A master plan that reserves a share of the housing stock for stable residential use, regulates changes of use in historic centres and protects functional diversity as a condition of foundational liveability changes the balance of power between those who extract rent and those who bear its costs. The Italian tradition of critical planning has always understood that planning is political; returning this awareness to international debate, starting from the concrete experience of Southern intermediate cities, is one of the contributions this article seeks to make.

## 7 Beyond the Italian case: scope conditions and a travelling research agenda

If the argument developed here has purchase beyond the three cities examined, it is because the mechanism it describes is not Italian but regime-specific. The conditions under which we would expect it to operate can be stated explicitly, which is the precondition for testing the framework elsewhere.

Four conditions appear to be jointly necessary. The first is a housing system in which property is widely and diffusely owned and the decommodified sector residual: this gives rent an interclass social base and converts its defence from a sectoral into a majoritarian interest. The second is a property market shallow enough to remain outside the circuits of institutional and financialized investment, so that valorization proceeds through a multitude of small operators rather than corporate strategies – less visible, harder to regulate, politically far better protected. The third is a productive base without alternatives: where tourism is one activity among several its costs can be weighed against other opportunities, whereas where it is the only available source of opportunity they are experienced as the price of vitality rather than as a harm to be contested. The fourth is a historic centre substantially emptied of residents in a previous cycle of expansion, whose devaluation constitutes the reservoir of the rent gap now being closed.

Where these four conditions hold, we would expect to find what we found: a rent gap that closes through the replacement of uses rather than of residents, an erosion of foundational liveability without significant displacement, and – the most counter-intuitive of our findings – a closure that generates consent rather than conflict. Southern Europe offers a large population of candidate cases, from the Andalusian and Galician provincial capitals to the Portuguese interior and the Greek regional centres. Central and Eastern Europe presents a particularly instructive variant: post-socialist restitution and mass privatization produced homeownership rates as high as those of the Mediterranean, but by a different historical route and without the same continuity between the construction cycle and local political power. Whether a real-estate bloc in Parlato’s sense can form on that basis is an open and, we think, decidable empirical question.

The negative cases matter as much. Where a substantial social-housing sector places an institutional floor beneath the conversion of stock to tourist use, the same pressure should produce a different distribution of its costs; where property is concentrated in corporate landlords, rent has a narrow social base and contestation should emerge earlier; and where no heritage assets generate tourist demand, the rent gap opened by twentieth-century expansion should simply remain open, producing devaluation without reinvestment. Each is a setting in which our argument makes a prediction that could fail.

Testing the framework comparatively requires measuring the right things, and this is perhaps the most immediately usable implication of our analysis. Research on intermediate cities has largely borrowed its indicators from the study of metropolitan gentrification, where the object of measurement is resident replacement. Those indicators are close to uninformative in the contexts we describe, in which population may be stable or rising while the residential function is eroded. What needs to be measured instead is use replacement, for which four families of indicators proved workable across all three cities and are constructible from data available throughout Southern Europe: the share of the residential stock withdrawn into short-term rental, and its concentration among multi-listing hosts, as a proxy for the professionalization of small property; the ratio between platform listings and officially registered accommodation, which measures the fraction of tourism rent that escapes public visibility; the ratio of hospitality to neighbourhood retail inside and outside the historic centre; and the floor area affordable at a fixed share of a locally representative wage, computed separately for purchase and for rent, which in our cases diverged and revealed a pressure that price indices alone conceal.

A final implication is methodological. The finding that the closure of the rent gap can be consensual is invisible to designs that infer conflict from structural pressure; it became available to us only because the survey and the interviews asked residents what they made of a transformation whose existence they clearly perceived. If consent rather than contestation is the modal response to tourism-led valorization in non-competitive cities, then the political question urban scholarship inherits from this literature – how rent is resisted – needs to be preceded by another: how it comes to be accepted.

## 8 Conclusion

This article has analysed urban transformations in three Italian intermediate cities – Cagliari, Catania and Lecce – using urban rent as the interpretive key to the dynamics of touristification, residential transformation and changing foundational liveability. Even in non-competitive contexts with relatively fragile property markets, rent continues to operate as a structuring principle of urban change, with profound effects on urban form, the residential function and the conditions of everyday life.

The hybrid and cumulative character of rent dynamics is the first finding. Marginal rent, linked to twentieth-century peripheral expansion, and differential rent, activated through the functional conversion of historic centres, are not distinct phases of urban development but interdependent processes: peripheral expansion emptied central spaces and thereby created the material conditions for their subsequent reactivation as sites of investment, consumption and tourism-led valorization. Touristification is not a rupture with previous trajectories but their contemporary reworking within the same regime of rent production.

The specificity of gentrification in intermediate cities is the second finding. The intensive models described in the classical literature operate in major metropolises; here gentrification is deferred and spatially selective, emerging where the residential function had already been weakened by previous cycles. What takes place is not direct social replacement but a progressive erosion of the conditions that make stable residence possible: rising housing costs, the withdrawal of dwellings from the ordinary market, the transformation of services, and the redefinition of the rhythms and uses of urban space.

The third finding concerns foundational liveability as a terrain of contradiction. In intermediate cities, liveability has historically been a distinctive feature and a symbolic resource, often mobilized in strategies of urban attractiveness; yet rent dynamics progressively undermine its material foundations across all three pillars. The crisis of residence, commercial specialization and coexistence conflicts are not unexpected side effects but structural outcomes of a development model based on tourism-led and property valorization in the absence of adequate regulation. Liveability is therefore not a technical issue to be managed through sectoral interventions; it is a political stake through which the question of who can effectively inhabit central places is redefined.

At the theoretical level, the article demonstrates the value of an approach that deconstructs the dominant metropolitan paradigm. Intermediate cities should be studied neither as marginal cases nor as miniature laboratories of metropolitan dynamics, but as contexts in which the contradictions of urban capitalism take specific – capillary, diffuse and politically rooted – forms. Analysing them requires categories of their own. Urban rent as a cross-cutting category, foundational liveability as a normative criterion, and the real-estate bloc as a tool for interpreting the politics of rent are contributions already developed by the Italian critical tradition that this article seeks to return to international circulation.

In terms of policy implications, the article’s message is more radical than its individual recommendations may suggest. Instruments regulating short-term rentals, housing policies oriented towards stable residence and measures protecting functional diversity are necessary. On their own, however – without intervention in the mechanisms of public rent capture, including land readjustment, taxation of value uplift and value-capture instruments (Micelli, 2021; Saiu and Blečić, 2021), and above all without the construction of a political coalition alternative to the real-estate bloc – they risk being little more than temporary dressings on an ever-widening wound. Major European cities are experimenting with forms of regulation aimed at curbing gentrification that would be unlikely to work in Southern intermediate cities, not because the available instruments differ, but because the social structure of rent does. Where ownership is widespread, small owners form part of the real-estate bloc and rent is the only significant form of accumulation, regulatory policies must be accompanied by redistributive ones that change the structure of interests rather than merely the rules of the game.

Beyond the comparative agenda set out in Section 7, further investigation of residents’ practices of resistance, adaptation and renegotiation would contribute to a more nuanced understanding of urban conflict in ordinary cities: we know how rent erodes foundational liveability, but still know little about how residents defend it.

## Data Availability

The datasets presented in this study can be found in online repositories. The names of the repository/repositories and accession number(s) can be found at: https://prin.unica.it/gc-lifes/research-output/.
